# Investigation of antibiotic resistance genotypic and phenotypic characteristics of marine aquaculture fish carried in the Dalian area of China

**DOI:** 10.3389/fmicb.2023.1222847

**Published:** 2023-06-23

**Authors:** Zihui Gao, Yongzhe Piao, Bing Hu, Chunhua Yang, Xiaobo Zhang, Qiuyue Zheng, Jijuan Cao

**Affiliations:** ^1^Key Laboratory of Biotechnology and Bioresources Utilization of Ministry of Education, College of Life Science, Dalian Minzu University, Dalian, China; ^2^Institute of Biological Resources, Jiangxi Academy of Sciences, Nanchang, Jiangxi, China

**Keywords:** Dalian, marine aquaculture fish, drug resistance, drug resistance genes, SYBG qPCR

## Abstract

Due to the long-term and irrational use of antibiotics for the prevention and control of bacterial diseases in aquaculture, antibiotic resistance genes have become a new source of pollution in aquatic products. Factors such as the spread of drug-resistant strains and the horizontal transfer of drug-resistant genes have led to multi-drug resistance in fish-infecting bacteria, which seriously affects the quality and safety of aquatic products. In this study, 50 samples of horse mackerel and puffer fish sold in Dalian aquatic products market and seafood supermarket were collected, and the phenotypic characteristics of the bacteria carried by the fish for drugs such as sulfonamides, amide alcohols, quinolones, aminoglycosides and tetracyclines were tested and analyzed, and the resistance genes carried by fish samples were detected by SYBG qPCR. Our statistical analyses demonstrated that the drug resistance phenotypes and genotypes of bacteria carried by mariculture horse mackerel and puffer fish in the Dalian area of China were complex, and the multi-drug resistance rate reached 80%. Among the examined antibiotics, the resistance rates to cotrimoxazole, tetracycline, chloramphenicol, ciprofloxacin, norfloxacin, levofloxacin, kanamycin, and florfenicol exceeded 50%, whereas the resistance rates to gentamicin and tobramycin were 26 and 16%, respectively. The detection rate of the drug resistance genes tetA, sul1, sul2, qnrA, qnrS, and floR exceeded 70% and all samples carried more than three drug resistance genes. The correlation analysis of drug resistance genes and drug resistance phenotypes showed that the detection of the drug resistance genes sul1, sul2, floR, and qnrD was correlated with the detection of drug resistance phenotypes (*p* < 0.01). However, the correlation between the resistance genes cmlA, cfr, tetA, qnrA, qnrS, and aac(6′)-Ib-cr and the corresponding resistance phenotype was not significant (*p* > 0.05). In general, our findings indicated that the multi-drug resistance of bacteria carried by marine horse mackerel and puffer fish in the Dalian area was serious. From the perspective of drug resistance rate and drug resistance gene detection rate, the aminoglycosides gentamicin and tobramycin are still considered effective in controlling bacterial infection in marine fish in the study area. Collectively, our findings provide a scientific basis for the management of drug use in mariculture, which can prevent the transmission of drug resistance through the food chain and minimize the associated human health risks.

## Introduction

Antibiotics are widely used to prevent and control bacterial infections in the medical, animal husbandry, and aquaculture fields, and can also be used as growth promoters in aquaculture, thus playing an important role in ensuring human health and the healthy development of aquaculture (Shi and Wang, 2018; Cheng et al., 2021). With the development of intensive farming patterns, large quantities of antibiotics are currently being used in aquaculture. Previous studies have estimated that global antibiotic consumption increased by approximately 69% between 2000 and 2015 and continues to grow at an annual rate of 4%. By frequency of use, the most commonly used antimicrobial classes were quinolones (27%), tetracyclines (20%), mycin (18%), and sulfonamides (14%) (Schar et al., 2020). In China, the world’s largest antibiotic producer and consumer, the total antibiotic use in 2015 alone reached 97,000 tons (Shi and Wang, 2018; Al Salah et al., 2019; Chen et al., 2020). Overuse and misuse of antibiotics have led to the rapid development of bacterial resistance, leading to increased healthcare costs, bacterial infections, and mortality (Du et al., 2022). In fact, more than 700,000 deaths worldwide each year are attributed to microbial drug resistance, and this figure is expected to increase to 10 million deaths by 2050 (Zhao et al., 2019; Sultan et al., 2020; Davtyan et al., 2021; Lai et al., 2021). The overuse and misuse of antibiotics in human disease control, as well as in the veterinary, agricultural, and aquaculture industries, can also lead to new sources of antibiotic resistance genes (ARGs), which may spread at higher rates between species through horizontal gene transfer (HGT), thereby exacerbating the problem of antibiotic resistance (Ye, 2020; Okeke et al., 2022). The ingestion of drug resistance genes carried by fish can lead to an imbalance of the normal flora in the human body, increase the resistance of pathogenic bacteria and conditionally pathogenic bacteria, and pose a serious threat to human health and disease prevention and control.

Antimicrobial susceptibility testing (AST) is a commonly used method for the detection of bacterial resistance, including paper diffusion, agar dilution, broth dilution, concentration gradients, and other classical methods (Jiang et al., 2022). For the detection of drug resistance genes, fluorescent probes, nucleic acid amplification, and high-throughput sequencing technologies can be used in conjunction with polymerase chain reaction (PCR) and quantitative reverse transcription PCR (RT-qPCR) to detect drug resistance genes (Nnadozie and Odume, 2019; Jiang et al., 2022). Among these approaches, paper diffusion is a simple and reproducible approach that does not require expensive equipment. However, it only provides phenotypic information about bacterial resistance and cannot detect resistance genes (Yalew, 2020; Jiang et al., 2022). The detection of drug resistance genes based on fluorescent probe technology has several advantages, including strong specificity. However, synthesizing fluorescent-labeled probes can be prohibitively costly. Moreover, high-throughput sequencing technology can be time-consuming, costly, and highly complex, in addition to lacking standardized and automated analysis processes, and is currently limited to pathogen diagnosis and screening of complex clinical diseases. In contrast, SYBG based qPCR methods are simple, time-saving, and cost-effective for rapid diagnosis and screening of clinical bacterial resistance genes (Jiang et al., 2022). Hossain et al. (2018) studied the resistance patterns of 43 strains of *Aeromonas* isolated from 46 zebrafish and the results showed that each isolate was resistant to at least four antibiotics, with multiple antibiotic resistance index values ranging from 0.22 to 0.50. Li et al. (2022) reported that nine species of freshwater fish and marine fish in a city in northern China contained four antibiotics, including doxycycline, tetracycline, sulfamethoxazole, and roxithromycin, in addition to 10 ARGs including strA. Hemamalini et al. (2022) reported that bacterial isolates in freshwater ornamental carp goldfish and tiger hooks had high resistance to bacitracin, rifampicin, trimethoprim, cephalexin, ampicillin, amoxicillin, nalidixic acid, and nitrofurantoin, and most bacterial isolates exhibited a multidrug resistance index of >0.2. Fauzi et al. (2021) used PCR and paper diffusion to detect drug resistance genes and their sensibility to 14 antibiotics in *Aeromonas* sp. isolated from 221 fish samples from Malaysia, and the results showed that the multiple antibiotic resistance index of the isolates ranged from 0.07 to 0.64, and resistance genes such as sul1, strA-strB, aadA, tetA-tetE, and tetM were detected. Khairy et al. (2019) evaluated vancomycin resistance using agar dilution and RT-qPCR and identified the VanB phenotype/vanA genotype in 33.3% vancomycin-resistant enterococcal isolates. Previous studies suggest that the detection of drug-resistant phenotypes and genotypes can be used to monitor drug-resistant bacterial infections and epidemics. However, few studies have investigated the correlation between bacterial resistance phenotypes and drug resistance genes. Liang Shaoshan et al. (Liao et al., 2022) investigated the drug resistance of *E. coli* in an aquaculture environment *via* the paper diffusion method and PCR method. The authors demonstrated that bacteria exhibited varying levels of drug resistance genes and drug resistance rates, but no specific analysis of the correlation between the two was conducted. Shen et al. (2022) tested antibiotic susceptibility and whole-genome sequencing in *Listeria monocytogenes* isolated from 1797 imported food samples collected between 2018 and 2020, and the analysis results demonstrated that the tetracycline- and chloramphenicol-resistant phenotypes were closely related to the genotype, whereas no clear relationship was identified between the remaining phenotypes and genotypes.

Dalian is an important city in the Bohai Rim region of China, whose primary aquaculture species are horse mackerel and pufferfish. This study analyzed samples of horse mackerel and puffer fish sold in Dalian aquatic products market and seafood supermarket were used as the research object, and the resistance of the samples to 10 antibiotics was determined through the antimicrobial-sensitive paper sheet method. SYBG qPCR was used to detect relevant resistance genes. Additionally, the correlation between the two methods was statistically analyzed and the consistency of drug resistance genes and drug resistance phenotypes was discussed. Finally, we assessed the applicability of drug resistance genes in drug resistance screening. The expression of drug resistance genes is regulated by a variety of substances and the causes of drug resistance are also affected by many factors. Therefore, it is important to study the correlation between bacterial drug resistance phenotypes and the detection of drug resistance genes. Here, we assessed the distribution and prevalence of antibiotic resistance and resistance genes in marine fish in Dalian, thus providing data support for the development of drug use strategies for aquaculture in Dalian. In turn, these measures could prevent the transmission of antibiotic resistance through the food chain and minimize their associated human health risks.

## Materials and methods

### Collection, storage and preparation of fish samples

Fifty fresh or frozen samples of horse mackerel and puffer fish were collected from Dalian aquatic products market and seafood supermarket, and the experimental fish samples collected were all healthy and disease-free, and each fish sample was individually packaged in a clean polyethylene bag, and transferred to an ice box and transported to the laboratory for storage at −20°C for later use.

### Sample pretreatment and DNA extraction

Under sterile conditions, after wiping the surface of the fish body with 70% alcohol for disinfection, 0.1–0.2 g homogenization was taken from the gill tissue sample, and placed in nutritional broth (Difco 240230 LB Broth, Lennox, BD Company, USA), and cultured at 36°C for 24 h for DNA extraction.

The Chelex 100 method was used to extract bacterial genomic DNA using the MightyPrep reagent for DNA kit (Code No. 9182; Baori Medical Biotechnology (Beijing) Co., Ltd) according to the manufacturer’s instructions. Next, 20 μL of bacterial solution was collected in 1.5 mL microcentrifuge tubes and 100 μL of the MightyPrep reagent for DNA reagent was added. The sample was then thoroughly mixed in a vortex shaker. Next, the samples were heated in a water bath at 95°C for 10 min, cooled to room temperature, and centrifuged at 12000 rpm in a Mini-15 K high-speed centrifuge (Hangzhou, Aosheng Instrument Co., Ltd.) for 2 min. The supernatant containing the DNA was then stored at −20°C for later use as a template for the PCR reactions.

### Drug resistance gene detection primer synthesis

This study used PCR primers for the analysis of 10 resistance genes for 5 classes of antibiotics, including the tetracycline resistance gene tetA; the sulfonamide resistance genes sul1 and sul2; the quinolone resistance genes qnrA, qnrD, and qnrS; the amide alcohol resistance genes cmlA, floR, and cfr; and the aminoglycoside resistance gene aac(6′)-Ib-cr. The primers were synthesized by Bao Bioengineering (Dalian) Co., Ltd., and the specific sequences are shown in Table 1.

**Table 1:** Primers for SYBG qPCR detection of drug resistance genes.

Types of antibiotics	Genes	Primer pair	Sequences (5′ → 3′)	Annealing temp. (°C)	Amplicon size (bp)	Reference
Sulfonamides	sul 1	FW	CGC ACC GGA AAC ATC GCT GCA C	62	163	Pei et al. (2006)
		RV	TGA AGT TCC GCC GCA AGG CTC G			
	sul 2	FW	TCC GGT GGA GGC CGG TAT CTG G	62	191	Pei et al. (2006)
		RV	CGG GAA TGC CAT CTG CCT TGA G			
Amide alcohols	cmlA	FW	GCC AGC AGT GCC GTT TAT	55	158	Li et al. (2013)
		RV	GGC CAC CTC CCA GTA GAA			
	floR	FW	CGG TCG GTA TTG TCT TCA CG	56	171	Li et al. (2013)
		RV	TCA CGG GCC ACG CTG TAT			
	cfr	FW	TGT GCT ACA GGC AAC ATT GGA T	55	148	Li et al. (2013)
		RV	CAA ATA CTT GAC GGT TGG CTA GA			
Quinolones	qnrA	FW	AGG ATT TCT CAC GCC AGG ATT	57	124	Vien le et al. (2012)
		RV	CCG CTT TCA ATG AAA CTG CA			
	qnrD	FW	AGT GAG TGT TTA GCT CAA GGA G	56.8	175	Vien le et al. (2012)
		RV	CAG TGC CAT TCC AGC GAT T			
	qnrS	FW	GTA TAG AGT TCC GTG CGT GTG A	54.6	189	Vien le et al. (2012)
		RV	GGT TCG TTC CTA TCC AGC GAT T			
Tetracyclines	tetA	FW	GCT ACA TCC TGC TTG CCT TC	62	210	Huang et al. (2015)
		RV	CAT AGA TCG CCG TGA AGA GG			
Aminoglycosides	aac(6′)-Ib-cr	FW	TGC GAT GCT CTA TGA GTG GCT A	55	482	Park et al. (2006)
		RV	CTC GAA TGC CTG GCG TGT TT			

FW, upstream primer; RV, downstream primers.

### qPCR detection of drug resistance gene

SYBG qPCR analysis was performed on a Flex QuantStudio TM 7 real-time fluorescence thermocycler (Thermo Fisher Scientific, USA). The amplifications were conducted in 25 μL reaction volumes containing 12.5 μL of TB Green Premix Ex Taq II (Tli RnaseH Plus) 2x, 1 μL of PCR Forward Primer (10 μM), 1 μL of PCR Reverse Primer (10 μM), 0.5 μL of ROX Reference Dye II (50×), 2 μL of DNA template, and 8 μL of DEPC treated water. The reaction consisted of a 30 s pre-denaturation step at 95°C, followed by 40 cycles of 95°C for 5 s and 60°C for 34 s, and finally 95°C for 15 s, 60°C for 1 min, and 95°C for 15 s. The presence of resistance genes was confirmed based on the fluorescence curve.

### Drug resistance phenotype detection

Drug susceptibility and resistance phenotyping were evaluated using the paper diffusion method (K-B) recommended by the American Committee for Clinical Laboratory Standardization (NC-CLS) (Ministry of Health of the People’s Republic of China, 2000). First, 1 mL of nutrient broth culture was evenly coated on three plates, after which 3–4 susceptibility paper sheets were adhered at equal intervals in the center of the plate. The plate with the antimicrobial susceptibility paper was then placed in a 36°C incubator for 16–18 h, after which the diameter of the antibacterial circle of the drug-sensitive paper was observed and recorded. *Escherichia coli* ATCC 35218 was used for testing and quality control. The antibiotic susceptibility paper tablets were purchased from Hangzhou Binhe Microbial Reagent Co., Ltd., China. A total of 10 antibiotics were tested in this study, including kanamycin (KAN, 30 μg/tablet), gentamicin (GEN, 10 μg ± 2.5 μg/tablet), tobramycin (TOB, 10 μg/tablet), ciprofloxacin (CIP, 5 μg/tablet), norfloxacin (NOR, 10 μg/tablet), levofloxacin (LEV, 5 μg/tablet), tetracycline (TET, 30 μg/tablet), florfenicol (FFC, 30 μg/tablet), chloramphenicol (CHL, 30 μg/tablet), and trimoxazole (SMZ, 25 μg/tablet). All statistical analyses for the drug resistance phenotypic data were conducted using the SPSS Statistics 17.0 software.

### Drug resistance spectrum analysis

Resistance spectra were analyzed against the Clinical and Laboratory Standards Institute (CLSI) antibiotic susceptibility testing standards (Clinical and Laboratory Standards Institute, 2011). According to the diameter of the antibiotic circle, the resistance of each antibiotic was classified as “S” (Sensitive), “I” (Intermediate), or “R” (Resistant), and each sample carried bacteria to form a unique resistance spectrum to the 10 antibiotics evaluated herein. The resistance spectrum was statistically classified, the drug resistance rate, multidrug resistance index, and drug resistance spectrum richness were calculated, and the resistance spectrum carried by the samples was plotted. The antibiotic resistance rate (ARR) refers to the ratio of the number of samples resistant to an antibiotic to the total number of samples tested. The multi-antibiotic resistance index (MARI) is the ratio of the number of antibiotics that a bacterial sample resisted from the 10 antibiotics tested in total. Resistance spectrum richness refers to the ratio of the number of resistance spectra of a sample to the total number of samples (Deng et al., 2020).

### Statistical analysis

For the fish samples collected in this study, the paper sheet method and the drug resistance gene method were used for drug resistance screening, and the diagnostic performance of the two methods in clinical samples was then discussed. The kappa values were calculated using the SPSS Statistics 17.0 software to assess method consistency. The kappa calculation results range from −1 to 1, and kappa values between 0 and 1 can be generally divided into five groups to represent different levels of consistency: 0.0–0.20, very low consistency (slight); 0.21–0.40, fair consistency (fair); 0.41–0.60, medium consistency (moderate); 0.61–0.80, high consistency (substantial); 0.81–1, almost completely consistent (nearly perfect).

## Results and analysis

### Resistance phenotype results

**Figure 1:**
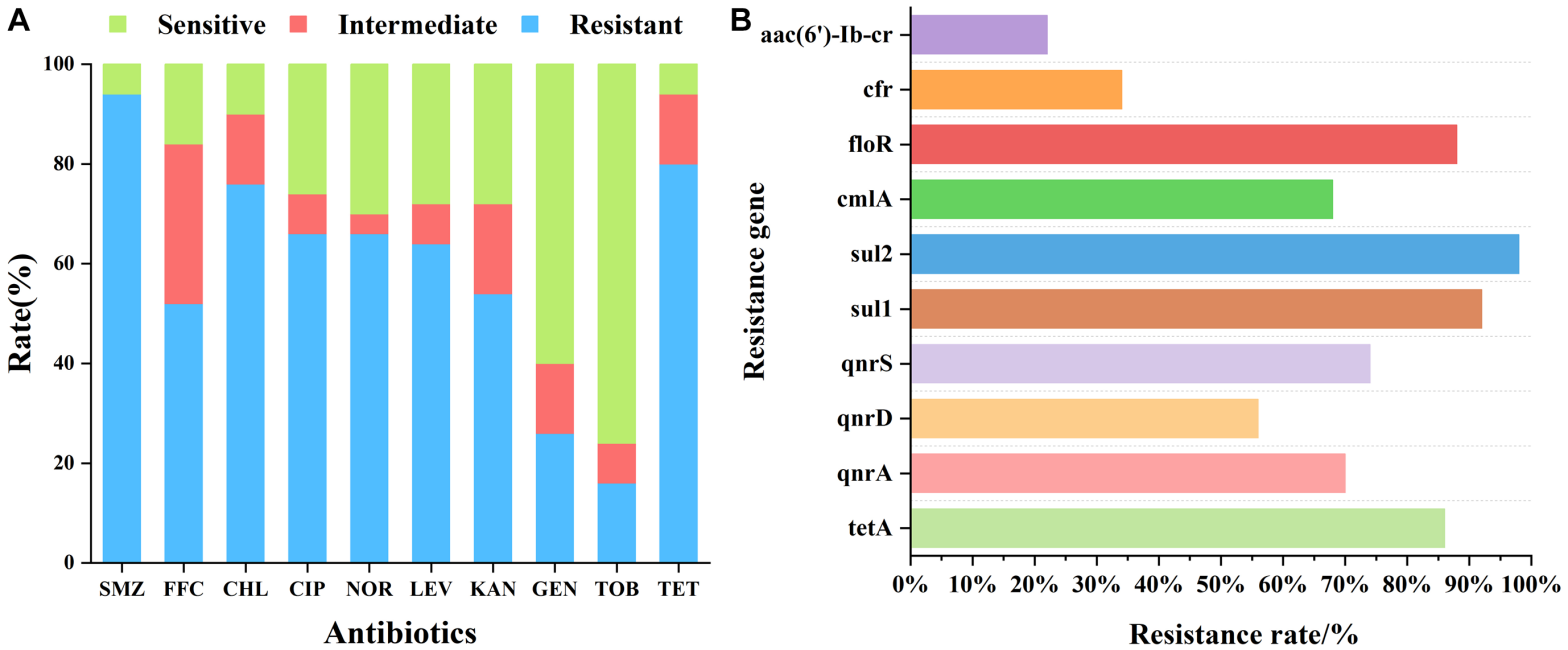
**(A)** Phenotypic screening of drug resistance in fish samples. The x-axis indicates the tested antibiotics and the y-axis indicates the resistance ratios of the fish samples to 10 antibiotics. **(B)** Detection of drug resistance genes in fish samples: the x-axis represents the detection rate of the corresponding resistance genes in all samples and the y-axis represents the resistance genes.

Fish samples were statistically analyzed for drug resistance to 10 antibiotics, and our findings revealed that the bacteria in the fish samples exhibited strong resistance to SMZ, TET, CHL, CIP, NOR, LEV, KAN, and FFC (drug resistance >50%), moderate resistance to GEN (25–50%), and low resistance to TOB (10–25%). Among the tested antibiotics, the bacteria exhibited a general resistance to sulfonamides and tetracyclines, with resistance rates of 94 and 80%, respectively. The resistance rates to the quinolones CIP and LEV were 66 and 64%, respectively, which were comparable to those of the banned NOR (66%). CHL is an amide alcohol drug whose application has been banned in aquaculture. However, its resistance rate was still 76%. FFC is an approved amide alcohol drug with an intermediate resistance of 32% and a resistance rate of 52%. The test samples were generally sensitive to aminoglycosides, except for KAN (54% resistance), GEN (26% resistance and 60% sensitivity), and TOB (16% resistance and 76% sensitivity). Table 2 and Figure 1A illustrate the patterns of resistance to 10 antibiotics in fish samples.

**Table 2:** Statistics of phenotype screening results of fish samples for drug resistance.

Types of antibiotics	Drug name (abbreviation)	Sensitivity (%)	Intermediary rate (%)	Drug resistance rate (%)
Sulfonamides	SMZ	6%	0%	94%
Amide alcohols	FFC	16%	32%	52%
	CHL	10%	14%	76%
Quinolones	CIP	26%	8%	66%
	NOR	30%	4%	66%
	LEV	28%	8%	64%
Aminoglycosides	KAN	28%	18%	54%
	GEN	60%	14%	26%
	TOB	76%	8%	16%
Tetracyclines	TET	6%	14%	80%

### Resistance gene results

The statistical analysis of our drug resistance gene detection results (Table 3; Figure 1B) indicated that the detection rate of drug resistance genes exhibited the following descending order: sul2 > sul1 > floR > tetA > qnrS > qnrA > cmlA > qnrD > cfr > aac(6′)-Ib-cr. Particularly, the frequency of detection of the tetA, sul1, sul2, qnrA, qnrS, and floR genes exceeded 70%. The detection rate of sulfonamide resistance genes sul1 and sul2 was also very high, reaching up to 90%, which was consistent with the 94% resistance rate of sulfonamides. The detection rate of the tetracycline tetA resistance gene was 86%, which was consistent with the detection rate of 80% of tetracycline resistance phenotypes. The floR gene is a florfenicol-specific resistance gene, and the detection rate for this gene was as high as 88%. This study focused on investigating the prevalence of quinolone qnr gene families, for which the detection rate exceeded 50%. Although only low-level quinolone resistance is mediated by the qnr genes, these genes can be horizontally transferred, thus accelerating the spread of quinolone-resistant strains and promoting the generation of resistant mutant strains. The detection rate of the aminoglycoside resistance gene aac(6′)-Ib-cr was 22%, which was consistent with the low detection rate of the aminoglycoside resistance phenotype.

**Table 3:** Detection of drug resistance genes.

Types of antibiotics	Drug resistance gene	Number of drug resistance gene detected samples	Drug resistance gene carrying rate (positive/total number of samples)
Sulfonamides	sul 1	46	92%(46/50)
	sul 2	49	98%(49/50)
Amide alcohols	cmlA	34	68%(34/50)
	floR	44	88%(44/50)
	cfr	17	34%(17/50)
Quinolones	qnrA	35	70%(35/50)
	qnrD	28	56%(28/50)
	qnrS	37	74%(37/50)
Aminoglycosides	aac(6′)-Ib-cr	11	22%(11/50)
Tetracyclines	tetA	43	86%(43/50)

### Drug resistance analysis of different species of fish samples

**Figure 2:**
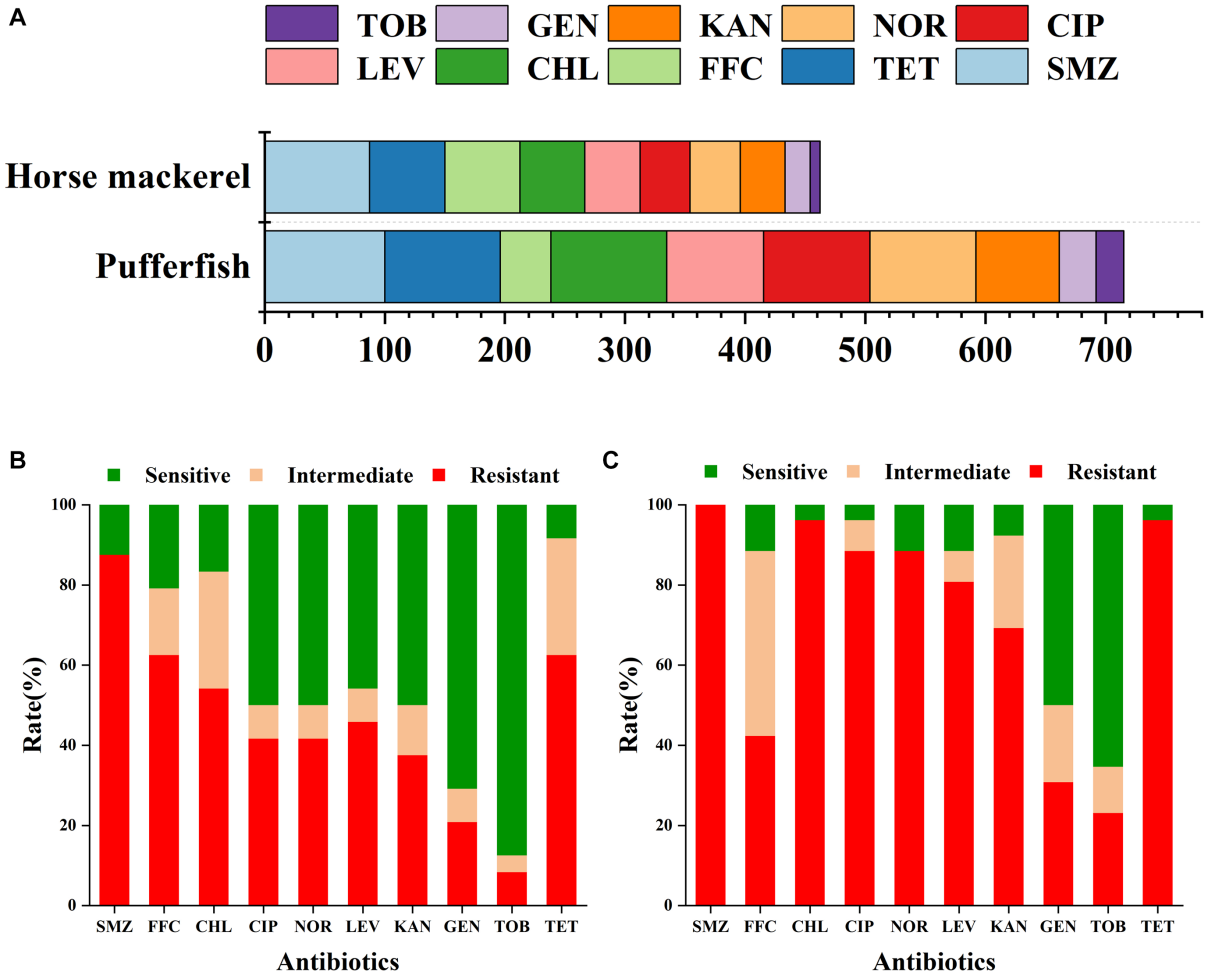
**(A)** Cumulative resistance rate of the ten examined antibiotics in the horse mackerel and pufferfish samples. Drug resistance of **(B)** horse mackerel and **(C)** pufferfish samples: the x-axis represents antibiotics whereas the y-axis represents the ratios of the resistance, mediation, and sensitivity of the two fish samples to 10 antibiotics.

Our study analyzed the resistance of pufferfish and horse mackerel bacterial samples to 10 antibiotics (Figure 2). The horse mackerel samples showed strong resistance to SMZ, TET, FFC, and CHL, with resistance rates of 87.5, 62.5, 62.5, and 54.17%, respectively. The samples also exhibited moderate resistance to LEV, CIP, NOR, and KAN, with resistance rates of 45.83, 41.67, 41.67, and 37.5%, respectively. Finally, we also detected low resistance rates to GEN and TOB of 20.83 and 8.33%, respectively. The pufferfish samples showed strong resistance to SMZ TET, CHL, CIP, NOR, LEV, and KAN, with resistance rates of 100, 96.15, 96.15, 88.46, 88.46, 80.77, and 69.23%, respectively; moderate resistance to FFC and GEN, with resistance rates of 42.31 and 30.77%, respectively; and a low TOB resistance rate of 23.08%. Both fish were sensitive to GEN and TOB, and the resistance and resistance rates were relatively consistent.

### Drug resistance spectrum analysis

**Figure 3:**
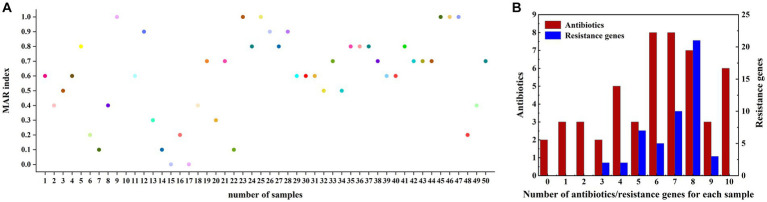
**(A)** Multidrug resistance MAR index plot of fish samples for 10 different antibiotics. **(B)** Multidrug resistance in fish samples; the x-axis represents the types of antibiotics tolerated or the number of resistance genes detected for each sample (including the detection of a minimum of 0 antibiotics or containing 0 resistance genes, and detecting a maximum of 10 antibiotics or containing 10 resistance genes). The left vertical axis represents the number of samples with a resistance phenotype detected (red histogram) and the right vertical axis represents the number of samples with resistance genes detected (blue histogram).

Our findings demonstrated that the examined fish samples carried complex drug-resistant phenotypes and genotypes, with 26 drug-resistant phenotypes and 33 drug-resistant genotypes. The multidrug resistance index (MARI) ranged from 0 to 1 and the richness of the resistance spectrum was 0.52. Figure 3A illustrated the distribution of the multidrug resistance index. All samples carried three or more resistance genes, with some carrying up to ten, of which eight were the main resistance genes, accounting for 42% (21/50). Two of the 50 samples were not resistant to any antibiotic and the remaining 48 samples were resistant to two or more antibiotics, some of which were resistant to all ten antibiotics. These 48 samples were predominantly resistant to six antibiotics and seven antibiotics, both with the same number of resistant samples, accounting for 16% of the total (8/50) (Figure 3B).

**Figure 4:**
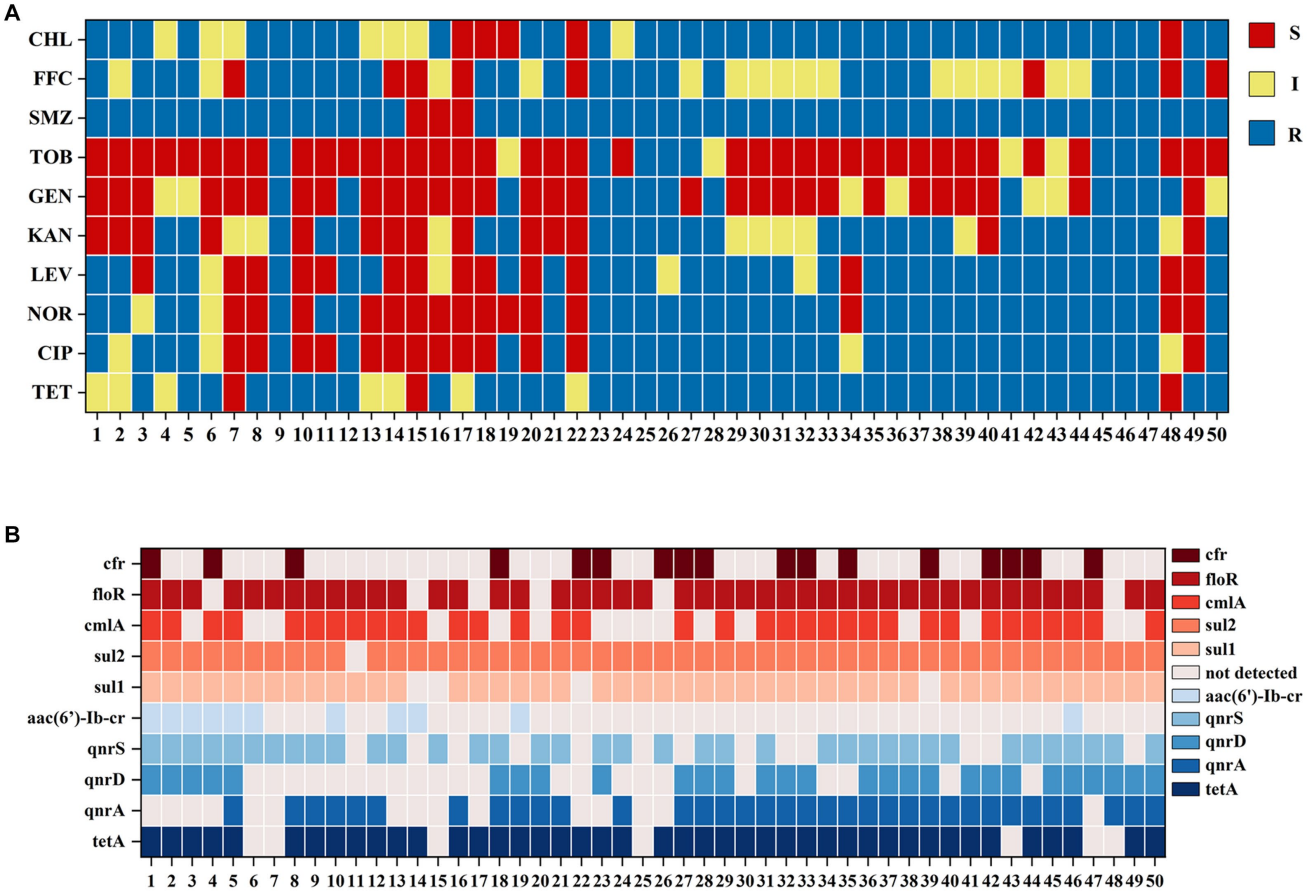
**(A)** Fish samples carrying bacterial resistance to drug-sensitive paper pieces. A total of 26 resistance phenotypes spectra were detected. **(B)** Fish samples carrying drug resistance genes were profiled. A total of 33 drug resistance genotypes were detected.

Statistical analysis of the carrying resistance phenotypes indicated that there were 26 resistance phenotypic spectra (Figure 4A), of which 6 samples were resistant to TET, CIP, NOR, LEV, KAN, GEN, TOB, SMZ, FFC, and CHL; 5 samples were resistant to TET, CIP, NOR, LEV, KAN, SMZ, CHL, TET, CIP, NOR, LEV, SMZ, and CHL; 4 samples were resistant to TET, CIP, NOR, LEV, KAN. SMZ, FFC, and CHL had 4 samples; 3 samples were resistant to SMZ and TET, SMZ, FFC, and CHL; 2 samples were resistant to TET, CIP, NOR, LEV, KAN, GEN, TOB, SMZ, FFC, and CHL; and the other 19 spectra had 1 sample each. Samples 17 and 19 were not resistant to any of the 10 antibiotics.

Statistical analysis of drug-resistant genotypes was conducted and a total of 33 drug-resistant genotypes were obtained (Figure 4B), of which 6 samples carried tetA, qnrA, qnrD, qnrS, sul1, sul2, cmlA, and floR; 5 samples carried tetA, qnrA, sul1, sul2, cmlA, floR; 4 samples carried tetA, qnrA, qnrD, sul1, sul2, cmlA, floR, and cfr; 3 samples carried tetA, qnrA, qnrS, sul1, sul2, cmlA, loR, and cfr; 2 samples carried tetA, qnrA, qnrD, qnrS, aac(6′)-Ib-cr, sul1, sul2, cmlA, floR; and 2 more samples carried tetA, qnrA, qnrD, qnrS, sul1, sul2, loR, and cfr and tetA, qnrA, sul1, sul2, and floR, respectively. The other 26 resistance genotypes had 1 sample each.

### Consistency analysis of drug resistance phenotype and drug resistance gene

The correlation between drug-resistant phenotypes and drug-resistant genes has recently garnered increasing attention among scholars. In this study, the kappa value was used to statistically analyze the consistency of the drug resistance phenotype and drug resistance gene detection results, as well as to explore the applicability of drug resistance genes in actual sample detection.

**Figure 5:**
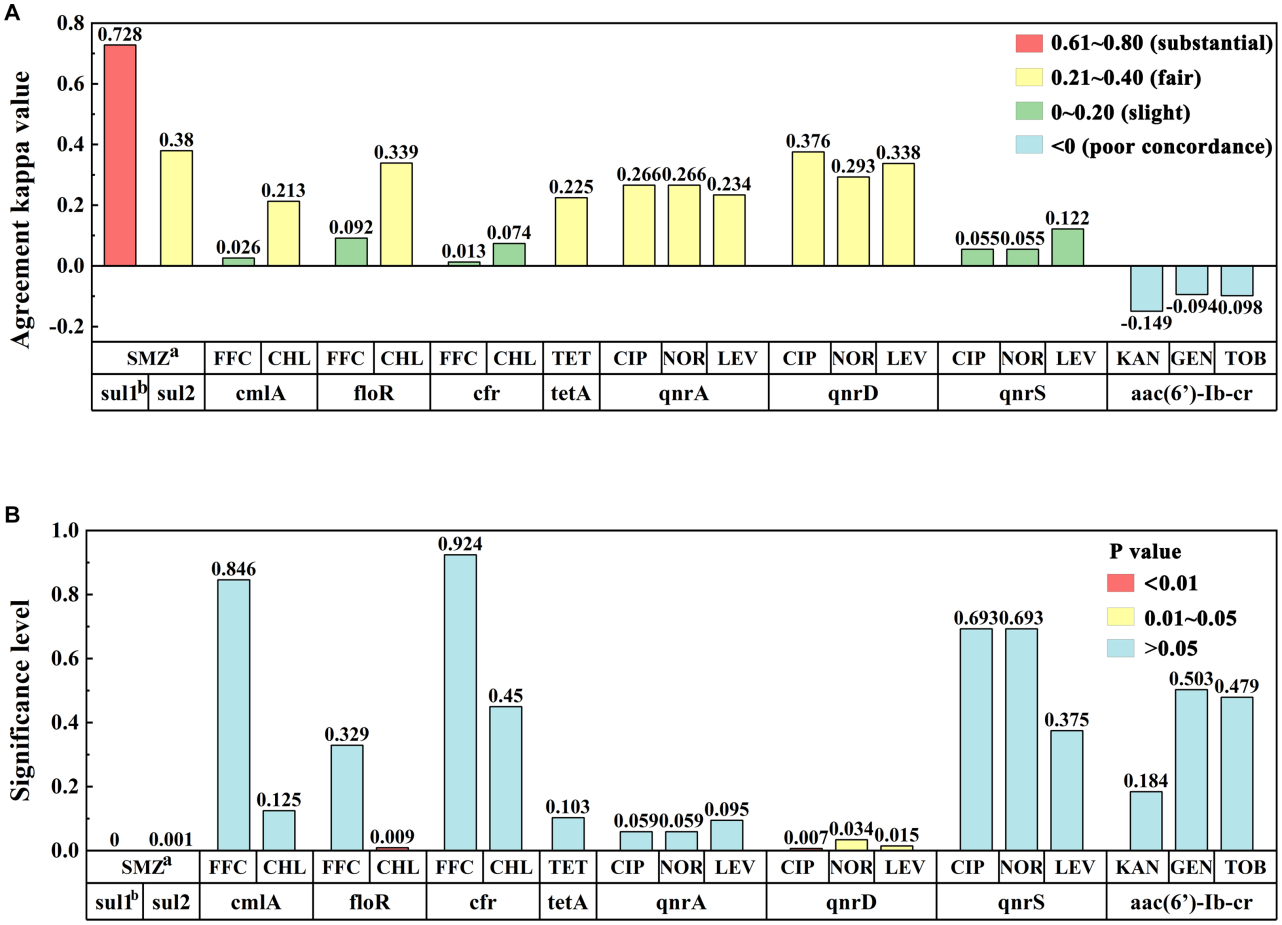
**(A)** Consistency analysis of kappa values between drug resistance phenotypes and drug resistance gene results. Row “a” represents antibiotics, row “b” represents resistance genes, and the y-axis represents the degree of consistency between the detection results of the two methods. **(B)** Significance analysis of drug resistance phenotype and resistance gene results: Row “a” represents antibiotics, row “b” represents resistance genes, and the y-axis represents significance levels between the test results of the two methods.

Statistical analysis of the kappa values between the drug-resistant genes and the susceptibility paper sheet method (Figure 5A) indicated that the kappa value of the alignment between the sul1 gene and the SMZ assay was 0.728, which was highly consistent between the two methods, and the kappa value of the alignment between the sul2 gene and the SMZ assay was 0.380, which was fairly consistent between the two methods, indicating that the sul1 gene could better reflect the resistance of sulfonamides. The consistency between the cmlA, floR, and cfr genes of amide alcohols and FFC was very low. The cmlA and floR genes were generally consistent with CHL, and the consistency of the cfr gene was very low with CHL. The tetracycline tetA gene was generally consistent with the TET phenotype. The quinolone qnrA and qnrD genes were generally consistent with CIP, NOR, and LEV, whereas the consistency of the qnrS genes was very low. The aminoglycoside aac(6′)-Ib-cr gene exhibited slightly less consistency with KAN, GEN, and TOB, which may be related to the complexity of the mechanism of aminoglycoside resistance.

Next, we conducted a correlation analysis of the drug resistance genes and the drug resistance phenotypic detection results (Figure 5B). The detection of drug resistance genes sul1, sul2, floR, and qnrD was correlated with the drug resistance phenotype of the sample (*p* < 0.05), whereas the correlation between the cmlA, cfr, tetA, qnrA, qnrS, aac(6′)-Ib-cr genes and their respective drug resistance phenotype was not obvious (*p* > 0.05). Among them, the sulfonamide resistance genes sul1 and sul2 were significantly correlated with the SMZ resistance phenotype (*p* < 0.01). There was a significant correlation between the floR gene and the CHL resistance phenotype (p < 0.01). For quinolones, the qnrD gene was associated with CIP, NOR, and LEV resistance phenotypes (p < 0.05). For tetracyclines and aminoglycosides, the tetA and aac(6′)-Ib-cr resistance genes carried by the samples were not associated with any resistance phenotypes.

## Discussion

Our findings demonstrated that drug resistance was serious and complex in marine aquaculture fish in Dalian, China. The test samples exhibited strong resistance to SMZ, TET, CHL, CIP, NOR, LEV, KAN, and FFC. Resistance to sulfonamides and tetracyclines was extremely high and therefore these antibiotics are not recommended in the study region. Although the use of CHL and NOR has been banned in aquaculture, these drugs still exhibited a relatively high resistance rate. This was presumably due to the high concentration of residues in the breeding environment caused by the indiscriminate use of these drugs, resulting in bacterial resistance. FFC is a broad-spectrum amide alcohol antibiotic that is currently used in mariculture to replace the banned drug CHL due to its low toxicity. This study examined whether CHL developed cross-resistance with FFC after banning, and our findings indicated that 21 of the 38 samples resistant to CHL also showed resistance to FFC, indicating that CHL-resistant bacteria also developed resistance to FFC. Additionally, our findings demonstrated that drug resistance to FFC was generally moderate (intermediary resistance rate of 32%). These findings suggest that the resistance to this drug gradually increased with increased administration, meaning that these drugs should be used with caution in mariculture. These findings were consistent with previous reports on the resistance of farmed fish to FFC in South China (Yin, 2022). The antibiotics with the lowest resistance rates among the test samples were the aminoglycosides GEN and TOB, and aminoglycoside resistance genes also had the lowest detection rates, indicating that the farmed fish in the study region were more sensitive to these drugs.

The correlation analysis of drug resistance phenotypes and drug resistance genes demonstrated that only the sulfonamide resistance genes sul1 and sul2, the chloramphenicol resistance gene floR, and the quinolone resistance gene qnrD were associated with the corresponding resistance phenotypes (*p* < 0.01), showing a good diagnostic consistency for resistance detection in clinical samples. Furthermore, our findings confirmed that the emergence of drug resistance is closely related to the occurrence of drug resistance genes. However, this relationship is not completely consistent. For example, the resistance phenotypes of multiple types of microbes did not show obvious correspondence with the resistance genes that they carry. These findings may be related to variations in the resistance mechanisms of each drug, as well as selection pressures in the environment. Several factors can inhibit the expression of resistance genes. However, ARG-carrying bacteria may have other potential mechanisms of drug resistance, thus highlighting the complexity of drug resistance in bacteria. Based on the drug resistance phenotypes and the detection rate of drug resistance genes elucidated in this study, marine aquaculture fish in the Bohai Sea area of Dalian exhibit strong antibiotic resistance and carry multiple drug resistance genes, which is closely related to the practice of intensive aquaculture. Therefore, other green prevention and control measures can be combined to focus on the use of aminoglycosides to control aquaculture diseases in the study region and reduce the use of other types of antibiotics. In turn, these measures could substantially decrease the expression of drug resistance genes and the horizontal spread of drug resistance to promote the healthy development of the aquaculture industry.

## Data availability statement

The datasets presented in this study can be found in online repositories. The names of the repository/repositories and accession number(s) can be found in the article/supplementary material.

## Author contributions

ZG conducted methodology, validation, investigation, writing-original draft, writing review and editing and the acquisition, analysis, and interpretation of data. YP and CY performed investigation and validation. BH performed sample detection. XZ performed actual sample detection. QZ performed conceptualization, supervision, funding acquisition, project administration, and substantively revised it. JC performed project administration and substantively revised it. All authors read and approved the final manuscript.

## Funding

The authors are greatful for the support of Dalian Science and Technology Innovation Fund (2022JJ13SN090), Dalian’s “Listing and Leading Plan” (XLYC2002106) and the High-Level Talent Innovation Project of Liaoning (XLYC2002106).

## Conflict of interest

The authors declare that the research was conducted in the absence of any commercial or financial relationships that could be construed as a potential conflict of interest.

## Publisher’s note

All claims expressed in this article are solely those of the authors and do not necessarily represent those of their affiliated organizations, or those of the publisher, the editors and the reviewers. Any product that may be evaluated in this article, or claim that may be made by its manufacturer, is not guaranteed or endorsed by the publisher.
